# Polyamine Catabolism Revisited: Acetylpolyamine Oxidase Plays a Minor Role Due to Low Expression

**DOI:** 10.3390/cells13131134

**Published:** 2024-07-01

**Authors:** Olga N. Ivanova, Anna V. Gavlina, Inna L. Karpenko, Martin A. Zenov, Svetlana S. Antseva, Natalia F. Zakirova, Vladimir T. Valuev-Elliston, George S. Krasnov, Irina T. Fedyakina, Pavel O. Vorobyev, Birke Bartosch, Sergey N. Kochetkov, Anastasiya V. Lipatova, Dmitry V. Yanvarev, Alexander V. Ivanov

**Affiliations:** 1 Engelhardt Institute of Molecular Biology, Russian Academy of Sciences, 119991 Moscow, Russiamartin.zenov@yandex.ru (M.A.Z.); nat_zakirova@mail.ru (N.F.Z.); pavel.gealbhain@gmail.com (P.O.V.);; 2 Gamaleya National Research Centre for Epidemiology and Microbiology, Ministry of Russia, 132098 Moscow, Russia; 3 INSERM U1052, CNRS UMR5286, Centre de Recherche en Cancérologie de Lyon, Université Claude Bernard Lyon 1, 69008 Lyon, France; 4 The Lyon Hepatology Institute EVEREST, 69003 Lyon, France

**Keywords:** polyamines, spermine, spermidine, acetylpolyamine oxidase, N^1^,N^11^-diethylnorspermine, virus replication, doxorubicin

## Abstract

Biogenic polyamines are ubiquitous compounds. Dysregulation of their metabolism is associated with the development of various pathologies, including cancer, hyperproliferative diseases, and infections. The canonical pathway of polyamine catabolism includes acetylation of spermine and spermidine and subsequent acetylpolyamine oxidase (PAOX)-mediated oxidation of acetylpolyamines (back-conversion) or their direct efflux from the cell. PAOX is considered to catalyze a non-rate-limiting catabolic step. Here, we show that PAOX transcription levels are extremely low in various tumor- and non-tumor cell lines and, in most cases, do not change in response to altered polyamine metabolism. Its enzymatic activity is undetectable in the majority of cell lines except for neuroblastoma and low passage glioblastoma cell lines. Treatment of A549 cells with N^1^,N^11^-diethylnorspermine leads to PAOX induction, but its contribution to polyamine catabolism remains moderate. We also describe two alternative enzyme isoforms and show that isoform 4 has diminished oxidase activity and isoform 2 is inactive. PAOX overexpression correlates with the resistance of cancer cells to genotoxic antitumor drugs, indicating that PAOX may be a useful therapeutic target. Finally, PAOX is dispensable for the replication of various viruses. These data suggest that a decrease in polyamine levels is achieved predominantly by the secretion of acetylated spermine and spermidine rather than by back-conversion.

## 1. Introduction

The biogenic polyamines spermine and spermidine are abundant oligocationic metabolites important for cell growth and differentiation [1,2]. Their roles in cells are mediated by interaction with DNA, RNA, and proteins, or by supporting spermidine-dependent posttranslational modification of protein(s)—hypusination, by scavenging reactive oxygen species, and other mechanisms. Spermidine was shown to act as a crucial regulator of longevity, which is linked to its ability to promote autophagy, mitophagy, and mitochondrial respiration [3]. Increased levels of polyamines are observed in various tumors and hyperproliferative pathologies, such as autoimmune diseases. Therefore, suppression of their biosynthesis or activation of catabolism is considered a promising strategy for the treatment of cancer and other hyperproliferative disorders. Initially, inhibitors of polyamine biosynthesis and inducers of their degradation showed no notable clinical effect in clinical trials in monotherapy regimes [4,5,6]. However, in recent years, progress has been made in treating primary and relapsed child neuroblastoma, providing evidence of the clinical importance of this metabolic pathway [7], although against other types of cancer, the efficacy remains insufficient [8].

Polyamines were discovered as early as the 17th century, when Antony van Leuwenhoek crystallized spermine [9]. Its structure was unveiled in 1926 [10]. The enzymes of polyamine metabolism were discovered during a period spanning from the late 1950s [11] to the early noughties [12,13,14]. The precursor of polyamines, 1,4-diaminobutane (putrescine, Put), is synthesized by ornithine decarboxylase (ODC) from the non-proteinogenic amino acid ornithine, a metabolite of the urea cycle [2,15]. This reaction is considered a rate-limiting step of polyamine biosynthesis. Then, an aminopropyl fragment is transferred from decarboxylated S-adenosylmethionine to the amino group of Put to produce spermidine, and then to the latter, yielding spermine. Polyamine degradation is achieved via one of two alternative pathways. First, both polyamines are subjected to acetylation by spermidine/spermine-N^1^-acetyl transferase (SSAT and SAT1) with either rapid oxidation by acetylpolyamine oxidase (PAOX) or efflux from a cell [2,16]. Then, spermine can be directly converted into spermidine by spermine oxidase (SMOX). Both oxidases, cloned only in 2001–2003 [12,13,14], generate a stoichiometric amount of hydrogen peroxide and 3-acetamidopropanal or 3-aminopropanal that are readily converted into highly toxic acrolein [17,18]. Polyamine oxidases and SMOX, in particular, are associated with the development of *Helicobacter pylori*- and *Bacteroides fragilis*-associated stomach and colon cancer [19,20] as well as with the pathogenesis of stroke [21].

However, this scheme has several blank areas [15]. One of them concerns the mechanism by which ODC is controlled by its natural inhibitor, a protein antizyme (OAZ). Antizyme is a protein that binds to ODC and targets it for proteasomal degradation. It also controls the influx of exogenous polyamines via a yet unidentified mechanism. The formation of active OAZ during high polyamine content is achieved via a frameshift event during the translation of its mRNA [22]. Only recently, Hyvonen et al. linked polyamine levels to OAZ biosynthesis and activity by showing that OAZ can undergo a spermine-/spermidine-dependent dimerization that affects both frameshift and its other functions [23].

Another neglected question in the polyamine field has been the contribution of PAOX to the catabolism of spermine and spermidine. The current assumption is that this enzyme is expressed constitutively, but that the rate of SSAT/PAOX activities in polyamine degradation depends solely on the expression of SSAT. Quantification of PAOX activity in various tissues is known to be heterogeneous, with the highest activity of the enzyme found in the pancreas and liver and the lowest activity in skeletal muscle [24]. Wallace and colleagues reported a decrease in PAOX expression in breast cancer compared to non-tumorous tissue [25]. There is also scarce evidence suggesting down-regulation of PAOX by genotoxic drugs [26]. Previously, our laboratory failed to detect PAOX gene transcription in human monocyte leukemia THP1 cells in the absence or presence of genotoxic stress triggered by the antitumor antibiotic doxorubicin [27]. Pledgie et al. demonstrated that upregulation of polyamine catabolism in mammalian cells led to enhanced production of H_2_O_2_ only via SMOX, not PAOX [28]. Actually, they detected neither PAOX activity nor mRNA. All these findings question the actual input of PAOX in polyamine catabolism in human cells. Additional questions arise from the different intracellular localizations of SSAT and PAOX: SSAT localizes to mitochondria [29] or the cytoplasm, probably at least in part due to its interaction with the α9 subunit of integrin α9β1 [30] or the diamine/amino acid transporter subunit SLC3A2 [31] on a plasma membrane, while PAOX localizes to peroxisomes [32]. These data imply that catabolism of spermine and spermidine via SSAT/PAOX requires acetylation in the cytoplasm/mitochondria, translocation of acetylated polyamines to peroxisomes for oxidation, and efflux of spermidine and putrescine back to the cytoplasm. This complex process is likely to be ineffective for such abundant metabolites as polyamines.

The first goal of this study was to re-evaluate the contribution of PAOX in polyamine catabolism by assessing its expression in various transformed and primary human cell lines and by analyzing the oxidation of endogenous and exogenous acetylated polyamines. An additional goal was to characterize the three isoforms of PAOX annotated in GenBank, of which two have not yet been described in the literature. The third goal was to analyze the role of PAOX in the replication of viruses and conferring resistance to anticancer drugs.

## 2. Materials and Methods

### 2.1. Reagents

Dansyl chloride, MDL72.527, spermine, spermidine, N^1^-acetylspermine, aminoguanidine, doxorubicin, cisplatin, temozolomide, and other reagents were from Sigma (Darmstadt, Germany). N^1^,N^11^-diethylnorspermine was purchased from Tocris Bioscience (Brystol, UK). Moreover, 2,2-Difluoromethylornithine (DFMO) was a kind gift from Prof. Patrick Woster (Medical University of South Carolina, Charleston, SC, USA). The pL4Puro vector was a gift from Prof. Peter Chumakov (Engelhardt institute of molecular biology, Moscow, Russia). pLP1, pLP2, and pVSV-G plasmids were from Invitrogen (Carlsbad, CA, USA).

A549 (CCL-185), K562 (CCL-243), HeLa (CCL-2), and SH-SY5Y (CRL-2266) cells were obtained from the American Tissue Culture Collection (ATCC, Manassas, VA, USA). DU145 cells were a kind gift from Dr. T. Keinanen (University of Eastern Finland, Kuopio, Finland). Huh7.5 cells were kindly provided by Prof. Charles Rice (The Rockefeller University, NY, USA) and Apath LLC (St. Louis, MO, USA). Low-passage cultures of primary glioblastoma multiforme (GBM4114, GBM6067, and GBM6138) from tumors removed surgically at the N.N. Burdenko Institute of Neurosurgery (Moscow, Russia) were described previously [33].

DMEM, MEM, and RPMI media were purchased from Gibco (Thermo Scientific, Waltham, MA, USA), and glutamine and trypsin-EDTA were purchased from PanEco (Moscow, Russia). Fetal bovine serum was supplied by HyClone (Logan, UT, USA). All oligonucleotides were synthesized by Evrogen (Moscow, Russia). Evrogen also provided Mint reverse transcriptase and the qPCRmix-HS SYBR mixture for real-time PCR.

### 2.2. RNAseq Data Analysis

We used NCBI SRA data to evaluate gene expression values for various human cell lines. The following datasets for human cell lines were analyzed: hepatocarcinoma HepG2 (PRJNA1090962, records SRR28422850, SRR28422851, and SRR28422849), hepatoma Huh7.5 and the differentiated into hepatocyte-like HepaRG cells (PRJNA494568) [34], primary human hepatocytes (PRJNA799365, records SRR17696837, SRR17696842, and SRR17696847), lung adenocarcinoma A549 cells (PRJNA559077, records SAMN12512941 and SAMN12512942) [35], osteosarcoma HOS cells, and glioblastoma multiforme DBTRG-05MG (GSE166877) [36], as well as primary glioblastoma cells derived from patients (GSE163949) [33]. Bioinformatic analysis of all these datasets was performed according to the procedures described in [34]. Read counts for each gene were presented as read counts per million (CPM). As housekeeping genes, β-actin (ACTB), glucuronidase β (GUSB), and glyceraldehyde-3-phosphate dehydrogenase (GAPDH) were used. As additional controls, monoamine oxidases A and B (MAOA, MAOB) and D-amino acid oxidase (DAO) levels were assessed.

### 2.3. Cell Culture Experiments

A549 cells were maintained in DMEM-F12 medium; Huh7.5, DU145, and glioblastoma cells were cultivated in DMEM; HeLa cells were cultivated in MEM, whereas K562 cells were cultivated in RPMI medium, all supplemented with 10% FBS and 2 mM glutamine. The cells were kept in a humid atmosphere of 5% CO_2_ at 37 °C and split every 3 days at a 1:3–1:4 ratio. Every 3–4 weeks, the cells were subjected to mycoplasma testing using the RT-PCR protocol. The cells were treated with 5 mM DFMO, 10 µM DENSpm, 30 µM MDL72.527, a mixture of spermine, and spermidine (100 µM each) supplemented with 1 mM aminoguanidine or 50 µM H_2_O_2_ if not stated otherwise.

### 2.4. RT-qPCR

Total RNA was purified from 0.5–1 × 10^6^ cells by the ExtractRNA reagent (Evrogen, Moscow, Russia) according to the manufacturer’s protocol, with subsequent treatment with the recombinant DNAse I (Roche, Basel, Switzerland). Reverse transcription was typically carried out using 500 ng of total RNA, a random hexamer primer, and Mint reverse transcriptase. Real-time PCR was performed as described previously [37], using the primers listed in Table 1.

### 2.5. Polyamine Quantification

The cells were grown on 10 cm plates to 92–100% confluence. After the removal of culture cell medium, the cells were washed with 3 mL of PBS at 0 °C and harvested using 1.5 mL of a 0.05% trypsin-EDTA solution with subsequent centrifugation (3200× *g*, 5 min at +4 °C). The pellet was resuspended in 2 mL of PBS, and the cells were harvested by centrifugation again. Then, they were resuspended in 1 mL of deionized water and disrupted by sonication in an ultrasonic bath (44 W/L, 3 min at 0 °C). The debris was removed by centrifugation (14,000× *g*, 10 min, 0 °C). The total protein content in supernatants was measured using the Pierce™ BCA Protein Assay Kit (Thermo Scientific, Waltham, MA, USA). Then, cellular proteins were removed by precipitation with the addition of 60% perchloric acid to a final 3% concentration and centrifugation (14,000× *g*, 10 min). The clarified lysates were lyophilized and kept at −80 °C prior to analysis.

Polyamines were quantified by HPLC with precolumn derivatization with dansyl chloride. The dry samples were dissolved in 20 µL water, neutralized by the addition of 40 µL 1.5 M NaOH, then 80 µL of saturated (at 20 °C) Na_2_CO_3_ solution, and the resulting mixtures were transferred to amber polyethylene microcentrifuge tubes. A mixture of 1,6-diaminohexane and 1,7-diaminoheptane was added as internal standards (0.2–5 nmol each, depending on the total protein concentration in a sample). The dansylation reactions were initiated by adding 40 µL of freshly dissolved dansyl chloride solution in dry dioxane (80 mg/mL), carried out in an ultrasonic bath at 40 °C for 10 min, and quenched by 2 µL of hydrazine hydrate. The reaction mixtures were vacuum dried at room temperature, resuspended in a mixture of 100 µL mQ and 250 µL toluene by vortexing for 1 min, and organic and aqueous phases were separated by centrifugation (10,000× *g*, 1 min). The toluene layer containing derivatized polyamines was removed from the new microtube, and the aqueous layer was re-extracted again with 200 µL of toluene. The toluene fractions were combined, vacuum dried, dissolved in 300 µL methanol, and subjected to HPLC analysis on a Cosmosil C18-MS-II, 250 × 4.6 mm, 5 µm (Nacalai Tesque, Kyoto, Japan) column heated at 40 °C under a pressure of 80–120 bar. The products were detected using a fluorescent flow unit at λex 340 nm and λem 530 nm. System A: 30% acetonitrile, 69.5% H_2_O, and 0.5% propionic acid. System B: 79.5% acetonitrile, 20% tetrahydrofuran, and 0.5% propionic acid. The elution gradient (1 mL/min): 0 min—0% B; 3 min—0% B; 62 min—70% B; 65 min—100% B, 70 min—100% B, 75 min—0% B, and 80 min—0% B.

Limits of detection (LOD) and quantification (LOQ) of this protocol were determined as 3:1 and 10:1 signal-to-noise ratio (S/N) by analyzing a mixture of polyamines and their acetyl derivatives in the range of 0.01–10 nmol/analysis, according to [38]. The reproducibility was determined by repeating the analysis by three different users in the laboratory and calculating the SD for each analyte from the pooled data. These values are provided in Supplementary Table S1.

### 2.6. Cloning of the PAOX Gene and Plasmid Construction

Total RNA was purified from DU145 cells using a High Pure RNA Isolation Kit (Roche). Two micrograms of the total RNA were subjected to reverse transcription using SuperScript IV (Invitrogen) and the (dT)_15_ primer. The PAOX1 open reading frame was amplified by nested PCR with primers 1 and 2 (Table 2) using Q5 DNA polymerase (New England Biolabs, Ipswich, MA, USA), followed by primers 3 and 4 using Phusion DNA polymerase in GC buffer (New England Biolabs). The final product was cloned into the EcoRI and XbaI sites of the pL4Puro lentiviral vector to give the pL4Puro-PAOX1 plasmid. A similar plasmid encoding the truncated PAOX4 isoform was obtained using the pre-phosphorylated primers 5 and 6 with Phusion DNA polymerase and ligation of the product into a circular construct. pL4Puro-PAOX2 was constructed similarly to primers 7 and 8.

To obtain plasmids for expression of PAOX1 in bacterial cells, the PAOX1 gene was amplified from the pL4Puro-PAOX1 construct using primers 9 and 10 with Q5 DNA polymerase and cloning of the product into EcoRI and XhoI sites of the two-cistron vector described previously for the expression of HCV RNA-dependent RNA polymerase [39]. Similar plasmids for the expression of PAOX2 and PAOX4 were constructed as described above for the lentiviral vector.

### 2.7. Stable Cell Lines

Recombinant lentiviruses encoding PAOX1, PAOX2, or PAOX4 were assembled in the HEK293T cells. Briefly, HEK293T cells were seeded on 10 cm well dishes in DMEM containing 10% FBS at a density of 3 × 10^6^/dish, 18 h later transfected by a mixture of 2.8 µg pLP1, 2.1 µg pLP2, 1.1 µg pVSV-G, and 2.8 µg of the corresponding pL4puro-PAOX plasmids using GenJect39, according to the vendor’s instructions. Twenty-four hours post-transfection, the medium was replaced with fresh, complete DMEM. Forty-eight hours post-transfection, the conditioned medium containing lentivirus particles was collected, filtered through a 0.45 µg syringe, and stored at −20 °C in aliquots.

Huh7.5 or A549 cells were seeded into 6-well plates at a density of 6 × 10^5^ cells/well and, on the next day, transduced with PAOX-encoding lentiviruses. Briefly, to each well, 2 mL of DMEM with 10% FBS containing 200 µL of the lentivirus stock and 22 µg polybrene was added, and 24 h later, this medium was replaced with the fresh one. Forty-eight hours post-transduction, the cells were split onto 10 cm dishes in DMEM with 10% FBS supplemented with 2 µg/mL puromycin for the selection of the transduced cells. Four days later, puromycin was removed, and when cells reached confluency, the expression of transgenes was analyzed by R-qPCR.

### 2.8. Expression and Purification of PAOX from Escherichia coli Cells

PAOX was expressed in the *Escherichia coli* Rosetta (DE3) strain. The cells transformed with the expression plasmids were grown in 400 mL Terrific Broth medium (TB) with ampicillin (100 μg/mL) and chloramphenicol (15 μg/mL) at 37 °C with shaking. When the optical density of the culture reached 0.5 (600 nm), the culture was cooled to 16 °C, isopropyl β-D-1-thiogalactopyranoside (IPTG) was added to a final concentration of 1 mM, and the cells were grown at 16 °C with vigorous shaking (300 rpm) for an additional 20 h. Afterwards, they were harvested by centrifugation (3200× *g*, 20 min), and the pellet was washed once with GTE buffer (25 mM Tris-HCl, pH 8.0, 50 mM glucose, 10 mM EDTA) and stored at −80 °C.

For purification of the enzyme, the pellet was thawn on ice, resuspended in 15 mL buffer A (25 mM Tris-HCl, pH 7.5, 500 mM NaCl, 10% (*v*/*v*) glycerol, 0.17 mg/mL phenylmethylsulfonyl fluoride (PMSF), and 0.1% of the protease inhibitor cocktail (Sigma, P8340) supplemented with 0.5% triton X-100, and lysed by sonication (80% power, 12 × 45 s intervals with a 1.5 min pause between them). Clarified by centrifugation (12,300× *g*, 30 min, 4 °C), lysate was applied onto a 0.3 mL Ni-NTA-agarose (Qiagen, Hilden, Germany) column at a speed of 1 mL/min. The column was washed with 15 mL of buffer A and then with the same buffer supplemented with 10, 20, 30, or 40 mM imidazole (10 mL for each fraction). The target protein was eluted by buffer A containing 250 mM imidazole, collecting fractions of 0.5 mL. The fractions containing the maximum amount of protein were pooled and dialyzed first against 500 mL buffer A (4 h, 4 °C) and then against 300 mL buffer B (25 mM Tris-HCl, pH 7.5, 100 mM NaCl, 10% (*v*/*v*) glycerol) (12 h, 4 °C).

The PAOX protein solution underwent anion-exchange chromatography (AKTA pure 25 L, GE Healthcare, Chicago, IL, USA) for further purification. The protein was applied on the FPLC Mono Q™ anion exchange chromatography column (1 mL), pre-equilibrated with 5 mL of buffer B, and eluted with a 300–1000 mM NaCl linear gradient in buffer B (21 mL) at 0.5 mL/min. PAOX was detected in the fractions containing. The fractions containing the maximum amount of PAOX (~500 mM NaCl) were pooled and dialyzed against 500 mL buffer C (25 mM sodium phosphate, pH 7.2, 100 mM NaCl, 10% (*v*/*v*) glycerol) for 12 h at 4 °C. The enzyme was stored at +4 °C. Protein concentration and purity were analyzed using electrophoresis in a 10% SDS-polyacrylamide gel with subsequent staining with Coumassie brilliant blue R-250 (Serva, Heidelberg, Germany).

### 2.9. PAOX Activity Assay

The cells were grown on 6 cm dishes, harvested by trypsin-EDTA and centrifugation at 4–6 °C, resuspended in 2 mL ice-cold PBS on ice, and collected by centrifugation again. Cell pellets were resuspended in 0.5 mL of 50 mM glycine-NaOH buffer (pH 9.5) and lysed upon incubation in an ultrasound bath at 40 W/L for 10 min at 0–4 °C. Debris was removed by centrifugation (10 min, 14,000× *g*, 4 °C); 80 µL aliquots were taken for quantification of total protein concentrations by the Pierce™ BCA Protein Assay Kit. The remaining lysates were aliquoted (100 µL); each aliquot was supplemented with 5 nmol N^1^-acetylspermine, and one aliquot was kept on ice (zero time point), while reaction mixtures were incubated for 30 min at 37 °C. The reactions were stopped by the simultaneous addition of 2 nmol 1,6-diaminohexane as an internal standard and chloric acid to a final concentration of 2%. The proteins were removed by centrifugation, clarified lysates were concentrated by two to three fold in speedvac, acid was neutralized with 1 M NaOH, and then dansylated and processed as described above. PAOX activity was calculated in 100 µL samples by quantifying the increase in its product (Spd) per minute normalized to the total protein amount.

### 2.10. Drug Toxicity Assay

Cells were seeded onto 96-well plates at a density of 3 × 10^4^ cells/well in DMEM supplemented with 10% FBS. On the next day, the medium was replaced with a fresh one containing doxorubicin, cisplatin, or temozolomide at various concentrations. Following 48 h of incubation, cell viability was assessed by a widely used MTT test [40].

### 2.11. Virus Replication Assays

#### 2.11.1. Influenza A and B Viruses

Influenza A (A/California/07/2009 pdm09) and B (B/Washington/02/2019 (B-Victoria Lineage)) strains were from the Russian Federation National Virus Collection at the National Research Center for Epidemiology and Microbiology named after Honorary Academician N.F. Gamaleya (Moscow). Influenza A and B viruses were cultivated in the allantoic cavities of 10-day-old chicken eggs at 36 °C for 48 and 72 h, respectively. Infectious and hemagglutination activities were quantified according to the protocols, as recommended by the World Health Organization (WHO) [41].

A549 and A549-PAOX1 cells were seeded onto 96-well plates at a density of 6 × 10^4^ cells/well in DMEM-F12 medium. They were washed with serum-free Eagle’s minimal essential medium (MEM) 24 h later, and the prediluted virus was added in 200 µL MEM at 10-fold serial dilutions. The virus titer was determined 96 h post-transfection by the evaluation of cytopathogenic effects, according to the Reed and Mench method [42].

#### 2.11.2. SARS-CoV-2

SARS-CoV-2 (passage 4) corresponding to the hCoV-19/Russia/Moscow-PMVL-12/2020 strain with an infectivity of 10^6^ TCID50/mL was obtained from the Russian State collection of viruses at the National Research Center for Epidemiology and Microbiology. Huh7.5 cells were seeded on 6 cm dishes at a density of 1 × 10^6^ in DMEM supplemented with 10% FBS. Twenty-four hours later, the medium was replaced with a medium containing SARS-CoV-2 at a multiplicity of infection (MOI) of 0.1 and 2.5% FBS. Two hours post-infection, the medium was replaced with fresh medium supplemented with 2.5% FBS. Virus RNA was isolated from conditioned medium, harvested 72 h.p.i., with the Probe-NK-S kit (DNA Technology, Moscow, Russia).

#### 2.11.3. Hepatitis C Virus (HCV)

A stock of HCV corresponding to the JFH1 isolate of the 2a virus subtype was prepared using a plasmid pJFH1 according to a standard protocol [43]. Highly permissive Huh7.5 cells, or Huh7.5-PAOX1 cells, were seeded on 6-well plates at a density of 3 × 10^5^ in DMEM supplemented with 10% FBS. Twenty-four hours later, the cells were infected with HCV at a MOI of 0.1, and at 6 h.p.i., the virus-containing medium was replaced with fresh medium. Six days post-infection, the cells were harvested, virus RNA was isolated using the kit for the purification of RNA on columns (Biolabmix, Novosibirsk, Russia), treated with recombinant DNase (Roche), reverse transcribed with Mint reverse transcriptase (Evrogen), and HCV RNA levels were quantified by qPCR according to the ΔΔct method.

#### 2.11.4. Other Viruses

Huh7.5 or Huh7.5-PAOX1 cells were seeded at a density of 2.5 × 10^4^ cells per well on 12-well plates in DMEM supplemented with 10% FBS and antibiotics. On the next day, the cells were infected with Sabin vaccine strain of type 1 poliovirus (PV1), Newcastle disease virus strain H2 (NDV), vaccine strain of mumps virus vaccine strain Leningrad-3 (MuV), oncolytic strain Moscow of mouse parainfluenza type 1 (SeV), and vaccinia virus Lister strain (VacV) in DMEM media at MOI 1. Two hours later, the cells were washed twice, and fresh media with 2% FBS was added. Forty-eight hours post-infection, cell supernatants were collected and titrated on sensitive cell cultures: H1299 for SeV and NDV, BHK21 for VacV, and HEK293T for PV1; viral titer was assessed by the standard Reed and Muench method [42]. In brief, cells were infected with 10-fold dilutions of supernatant, and after 72 h, cell viability was assessed, viral titer concentration was quantified.

### 2.12. Statistical Analysis

Statistical analysis was carried out using GraphPad Prism 7 (GraphPad Software LLC, Boston, MA, USA). All data are presented as mean values ± standard deviation (S.D.) of three independent experiments, if not stated otherwise. Differences between two groups were compared using a two-tailed unpaired or paired Student’s *t*-test. For comparison between multiple groups, a mixed ANOVA followed by a Sidak’s post-hoc test was applied. *p*-values < 0.05 were considered statistically significant if not stated otherwise.

## 3. Results

### 3.1. PAOX Expression Is Low in Various Human Cell Lines Based on RNA-Seq Data from NCBI SRA

**Figure 1:**
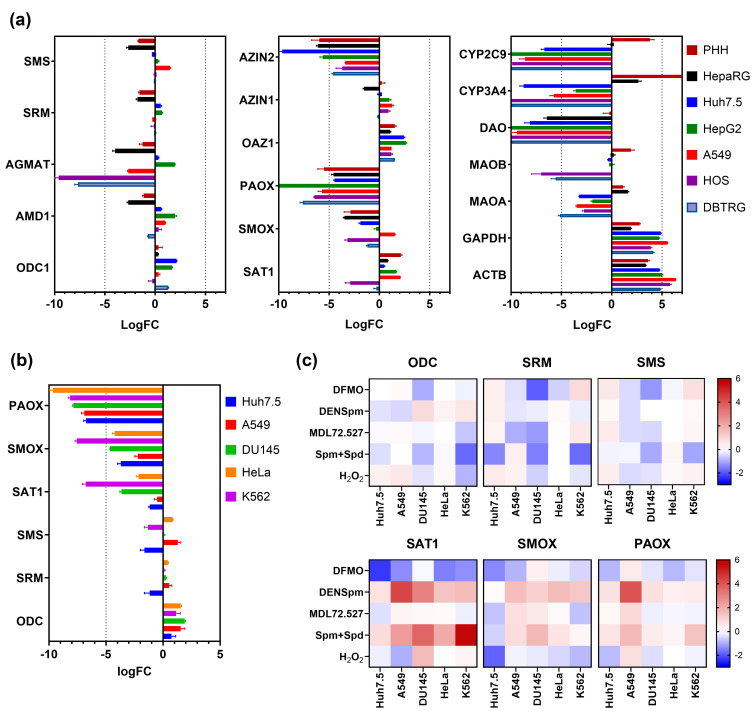
Transcription of genes encoding polyamine-metabolizing proteins in tumor- and non-tumor cell lines. (**a**) RNA-seq data depicting relative levels of mRNAs encoding proteins of polyamine metabolism and other proteins in tumor Huh7.5, HepG2, A549, HOS, and DBTRG cell lines as well as in non-tumor primary human hepatocytes (PHH) and hepatocyte-like HepaRG cells, normalized to levels of GUS mRNA and represented as binary logarithms of fold change LogFC (n = 3 for all datasets with the exception of data for A549 cells—n = 2). (**b**) Quantification of relative mRNA levels encoding enzymes and regulatory proteins of polyamine metabolism in cell lines by RT-qPCR, normalized to the levels of GUS mRNA (n = 3). (**c**) Relative levels of mRNAs encoding polyamine-metabolizing enzymes, normalized to GUS mRNA levels and to levels in untreated cells and expressed in Log2FC. Data are shown as means ± SD.

When quantifying expression of various metabolic genes in different cell lines by RT-qPCR, we noted that the levels of PAOX mRNA were the lowest among the levels of mRNA of various polyamine-metabolizing enzymes and regulatory proteins and almost always remained close to the limit of detection by SYBR Green-based RT-qPCR. Therefore, our first goal was to perform a systematic re-assessment of PAOX expression and role in polyamine catabolism in various human cell lines. Our first approach included the analysis of several datasets of RNA-seq data obtained by us previously or reported by other groups. They included datasets based on human hepatoma Huh7.5 cells and differentiated non-transformed HepaRG cells, which upon differentiation contain hepatocyte- and cholangiocyte-like cells (PRJNA494568) [34]. We also used recently obtained data from HepG2 cells (deposited as PRJNA1090962), human glioblastoma multiforma DBTRG-05MG, osteosarcoma HOS cells (GSE166877), and lung adenocarcinoma A549 cells (PRJNA559077). We analyzed levels of the biosynthetic enzymes ODC, S-adenosylmethionine decarboxylase (AMD1), spermidine, spermine syntases (SRM and SMS), and agmatinase (AGMAT). We also assessed levels of catabolic enzymes (SAT1, SMOX, and PAOX) and regulatory proteins (OAZ and antizyme inhibitors—AZIN). The expression values were presented as average read counts per million (CPM) and compared to the corresponding values for reference genes. We did not use standard RNA-Seq normalization techniques (TMM, RLE, etc.) in order to better compare the data with RT-qPCR, which uses reference genes. As such, β-glucoronidase (GUS), β-actin, and GAPDH were included in the analysis. Finally, as controls of low-expressing genes, we included (i) amino oxidases 1 and 2 that are readily expressed in various tissues with low-to-moderate expression in most cell lines, (ii) D-amino acid oxidase (DAO) that is not expressed in human tissues except for some minor levels in the CNS system, and (iii) cytochromes P450 (CYP) isoforms 3A4 and 2C9 that are expressed only in mature hepatocytes. An additional rationale for the analysis of MAO and DAO expression was the fact that these enzymes could be involved in the catabolism of putrescine and some acetylated polyamines [44]. Figure 1a clearly shows that the levels of PAOX gene transcription are the lowest among various polyamine-metabolizing enzymes and regulatory proteins.

Next, we evaluated transcription of genes encoding PAOX and other polyamine-metabolizing proteins by RT-qPCR in several human cell lines, including Huh7.5 (hepatoma), A549 (lung adenocarcinoma), DU145 (prostate carcinoma), HeLa (cervix adenocarcinoma), and K562 (chronic myeloid leukemia). DU145 was chosen as this line is widely used in the polyamine field, and HeLa—since this cell line has been reported to exhibit the highest levels of PAOX gene expression, according to HIPED (the Human Integrated Protein Expression Database) [45]. mRNA levels normalized to GUS, the housekeeping gene), are presented in Figure 1b. Again, PAOX mRNA levels were the lowest among all transcripts of polyamine-metabolizing enzymes. On average, they were two orders of magnitude lower than SRM mRNA levels or almost 400-fold lower than ODC mRNA levels.

### 3.2. Altered Polyamine Metabolism Has a Low Impact on Levels of PAOX mRNA

The observed low levels of PAOX expression could have resulted from the absence of stimulation by an unidentified factor(s). We, therefore, re-evaluated the impact of various factors on PAOX gene transcription, including (i) inhibition of polyamine biosynthesis with 2,2-difluoromethylornithine (DFMO), (ii) activation of catabolism with N^1^,N^11^-diethylnorspemine (DENSpm) that induces SAT1 and SMOX, (iii) shifting the spermidine/spermine ratio by treatment with MDL72.527, an inhibitor of SMOX and PAOX; (iv) elevation of the polyamine pool via the incubation of cells with spermine, spermidine, and aminoguanidine as an inhibitor of serum aminooxidases; and (v) H_2_O_2_-triggered oxidative stress. Most stimuli led to decreased transcription of genes that encode biosynthetic enzymes (ODC, SRM, and SMS) (Figure 1c). DENSpm and exogenous polyamines induced transcription of SAT1 and, to a lesser extent, of SMOX and PAOX. We should point out that changes in PAOX and SMOX mRNA levels were generally moderate. The only pronounced effect on PAOX transcription was observed in lung adenocarcinoma A549 cells, while in Huh7.5, HeLa, and DU145 cells, no effect of any of the stimuli was observed. It is also worth noting that hepatoma Huh7.5 cells were resistant to most stimuli for all polyamine-metabolizing enzymes.

### 3.3. PAOX Activity Is Undetectable in Most Cell Lines, except a Few Glioblastoma Cell Lines

**Figure 2:**
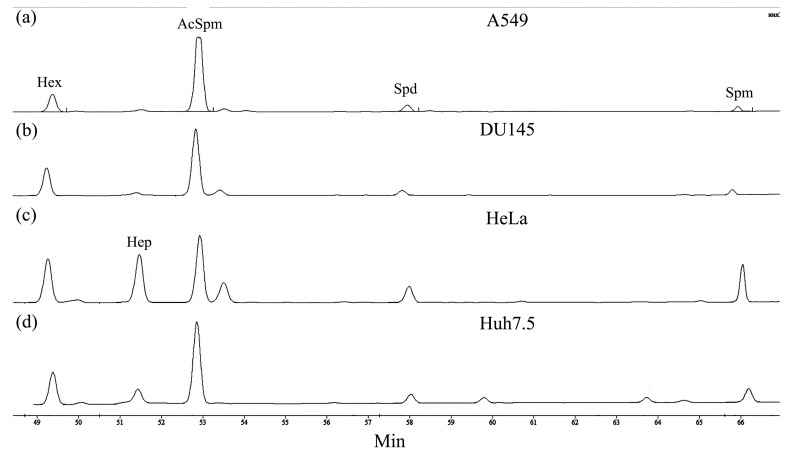
PAOX activity is below the limit of detection in various human cell lines. A549 (**a**), DU145 (**b**), HeLa (**c**), and Huh7.5 (**d**) cells were lysed on ice; 5 nmol of N^1^-acetylspermine (AcSpm) was added to the clarified lysates that were kept at 37 °C for 30 min; and the possible conversion of AcSpm into spermidine (Spd) was monitored by HPLC analysis. Then, 1,6-Diaminohexane (Hex) and 1,7-diaminoheptane (Hep) were added to reaction mixtures as internal standards.

To address the question of whether low transcription levels could still ensure the production of a notable amount of functional enzyme, PAOX enzymatic activity was measured in cell lysates supplemented with exogenous N^1^-acetylspermine (AcSpm). This reaction was monitored by HPLC analysis. The elution profiles contained peaks for exogenous AcSpm, endogenous Spd and Spm, and 1,6-diaminohexane and 1,7-diaminoheptane that were added as internal controls (Figure 2, Supplementary Figures S1–S3 and Table S2). In all these cases, Spm and Spd peak intensities were comparable. PAOX activity would be expected to decrease the intensity of the AcSpm peak (substrate) and increase the intensity of the Spd peak (product). However, during the 30 min incubation period, no conversion of AcSpm into Spd was observed, suggesting the absence of noticeable PAOX activity in A549, DU145, HeLa, and Huh7.5 cells. And unfortunately, we would not verify these data using commercial anti-PAOX antibodies that did not stain even the recombinant enzyme.

**Figure 3:**
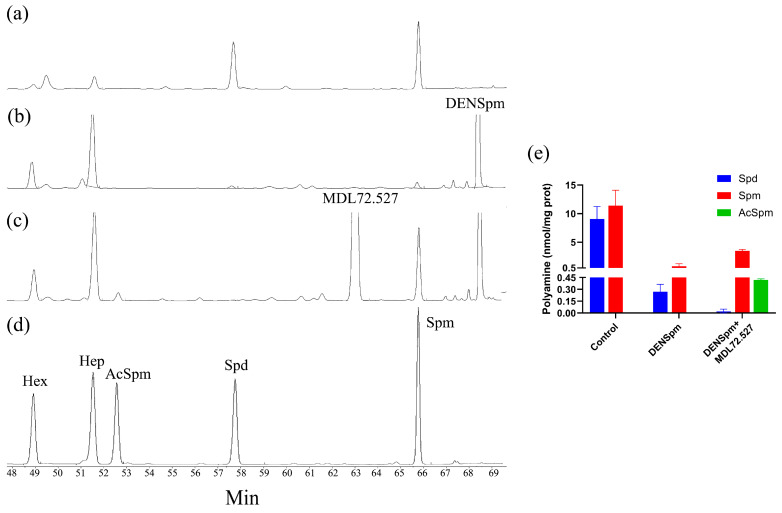
In A549 cells treated with DENSpm, PAOX is expressed but plays only a minor role in polyamine catabolism. Polyamine levels were analyzed by HPLC in untreated A549 cells (**a**), treated with 10 µM DENSpm alone (**b**), or together with 50 µM MDL72.527 (**c**), and the results of quantification are presented as means ± S.D. (n = 3) (**e**). Panel (**d**) represents the elution profile for a control mixture of spermine (Spm), spermidine (Spd), and N1-acetylspermine (AcSpm), as well as 1,6-diaminohexane (Hex) and 1,7-diaminoheptane (Hep) used as internal standards. All chromatograms are representative of three independent analyses.

An alternative approach was to induce the production of acetylated polyamines in cells with DENSpm and to assess the role of PAOX in their degradation with MDL72.527, an irreversible inhibitor of both polyamine oxidases. This was carried out in A549 cells, as in this particular cell line, there was an induction of PAOX mRNA, as discussed above. Indeed, DENSpm caused a dramatic decrease in intracellular levels of spermine and spermidine, with their total concentrations less than 5% of those in untreated cells (Figure 3). Notably, N^1^-acetylspermine was not detected in these samples. Additional treatment with MDL72.527 led to a further decrease in Spd and partial restoration of Spm levels, which were likely due to inhibition of SMOX. Moreover, MDL72.527 allowed us to detect AcSpm, thus supporting the presence of PAOX activity in A549 cells during DENSpm treatment. As the increase in AcSpm was several folds lower than that of Spm, one can conclude that PAOX activity in these cells is lower than that of SMOX (Figure 3e). And as the total pool of polyamines in cells treated with both drugs was still five-fold lower than in control cells, the export of acetylated polyamines played a significant role in the exhaustion of the polyamine pool during DENSpm treatment. All this speaks against the predominant role of PAOX in polyamine catabolism in this cell line.

### 3.4. PAOX Activity Can Be Detectible in Glioblastoma and Neuroblastoma Cell Lines with High Polyamine Levels

We also continued searching for cell lines that express PAOX at levels sufficient for the detection of enzymatic activity. We analyzed SH-SY5Y neuroblastoma cells as well as three low-passage glioblastoma cell lines obtained previously from patients: GBM4114, GBM6067, and GBM6138. When these lysates were incubated with exogenous acetylspermine, we registered a decrease in AcSpm and an accumulation of Spd. The data expressed as rates of Spd synthesis per min per mg of total protein are presented in Table 3. Thus, in contrast to the cell lines used above, glioblastoma and neuroblastoma cell lines exhibit detectable PAOX activity. The highest rate was registered for the GBM6138 cell line.

To reveal the differences between cell lines with visible PAOX activity and PAOX-negative cell lines, levels of polyamines were quantified. As is clearly seen from Table 3, glioblastoma cells, with the exception of GBM6067, exhibit a markedly higher concentration of spermine and, to some extent, spermidine. However, in HeLa cells (PAOX-negative), the polyamine levels were also high. Nevertheless, it could be concluded that PAOX is expressed in cells with profoundly elevated polyamine pools.

### 3.5. Alternative PAOX Isoforms Have Impaired Catalytic Activity

**Figure 4:**
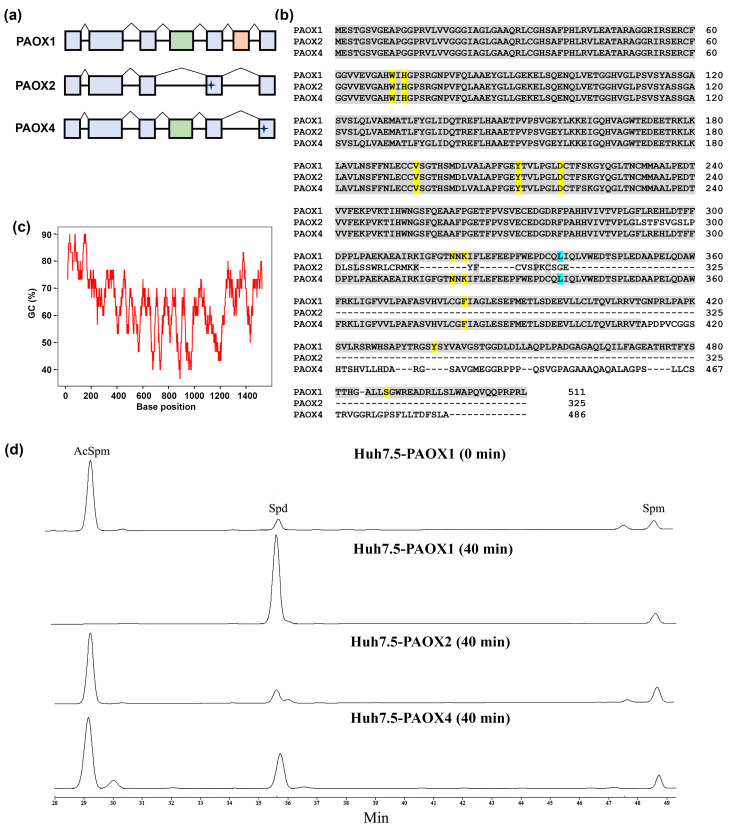
Alternative PAOX isoforms have diminished enzymatic activity. (**a**) Comparison of open reading frames of PAOX1, PAOX4, and PAOX5 mRNAs. Exons present in all isoforms are in light blue; the exons that are missing in short isoforms are in green and orange. (**b**) Alignment of PAOX1, PAOX2, and PAOX4 sequences. Amino acid residues of PAOX1 and the same residues of alternative isoforms are given in gray. Amino acid residues that are critical for substrate binding and catalysis and are conserved between human and murine PAOX—in yellow and homologous—in blue. (**c**) Analysis of GC content in the protein coding region of PAOX1 mRNA. (**d**) Representative HPLC profiles showing the conversion of AcSpm into Spd by PAOX isoforms.

Though in the scientific literature PAOX is described as a sole isoform of the protein composed of 511 amino acids, there are three variants of its mRNA in Genbank: 1 (NM_152911), 4 (NM_207127), and 5 (NM_207128) (Figure 4a and Table 4). Variants 4 and 5 of mRNA encode truncated isoforms of the protein, denoted as isoform 2 (325 amino acid residues, NP_997010) and 4 (486 amino acid residues, NP_997011), respectively (Figure 4b, Table 2). To the best of our knowledge, their expression in cells and functionality have never been evaluated. So, our next goal was to clone and measure the enzymatic activity of these three PAOX variants. As the PAOX gene has a very high GC content, reaching 80–90% in its N-terminal part (Figure 4c), the gene was cloned using Q5 DNA polymerase from the total RNA of DU145 cells. Noteworthy, the cloning yielded only isoform 1, while no signs of expression of alternative isoforms were found. The isoforms 2 and 4, representing the truncated variants, were constructed by PCR-based subcloning. All three variants of the gene were cloned into a vector for expression in bacteria and into a lentiviral vector for subsequent expression in mammalian cells. Our attempts to compare the enzymatic activity of recombinant proteins expressed and purified from *E. coli* failed, as isoforms 2 and 4 formed inclusion bodies and could not be purified in non-denaturing conditions. Analysis of intracellular PAOX activity in Huh7.5 cell lines transduced with PAOX1, PAOX2, and PAOX4-encoding lentiviruses was performed by monitoring the conversion of exogenous N^1^-acetylspermine into spermidine in cell lysates. Indeed, PAOX1 was highly active, as the rate of Spd synthesis in the lysate of Huh7.5-PAOX1 cells supplemented with AcSpm was 2253 pmol/min*mg of total protein (Figure 4d). The rate of conversion was approximately three-fold higher than in the glioblastoma GBM6138 cell line. In contrast, PAOX2 was inactive, as the level of Spd synthesis from exogenous AcSpm in Huh7.5-PAOX2 cell lysate was below the limit of detection (<25 pmol/min*mg total protein). Finally, PAOX4 exhibited very low activity: 65.6 pmol/min*mg total protein).

### 3.6. PAOX Is Dispensable for the Replication of RNA Viruses

Polyamines are indispensable for the replication of various DNA and RNA viruses (reviewed in [2,46]). Inhibitors of polyamine biosynthesis and of hypusination (a spermidine-dependent type of post-translational modification) act as broad-range antiviral agents [47,48,49]. Moreover, MDL72.527, the inhibitor of PAOX and SMOX, was reported to exhibit antiviral activity towards SARS-CoV-2, Ebola, and hepatitis C virus (HCV) [49,50,51], implying that at least one of these oxidases is critical for the replication of viruses. So, our next goal was to investigate the role of PAOX in the replication of DNA and RNA viruses. We used two tumor cell lines for the following reasons. First, our main object, HCV, replicates only in the human hepatoma Huh7.5 cell line [52,53]. Besides, anti-HCV activity of MDL72.527 was also reported in this cell line [51]. Second, SARS-CoV-2 infection also replicates in Huh7.5 cells with no apparent cytopathic effect, which has exhibited a non-specific effect on polyamine metabolism.

**Figure 5:**
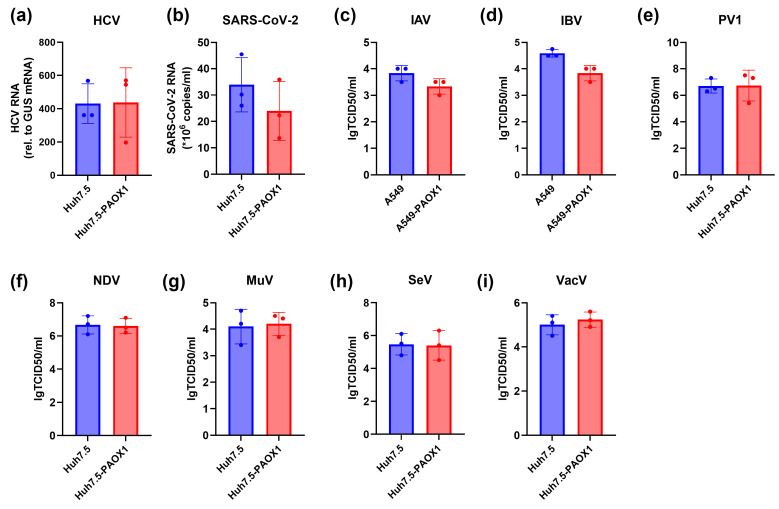
Overexpression of PAOX1 does not affect the replication of RNA and DNA viruses. Naïve Huh7.5 or A549 cells (blue bars) or the cells overexpressing PAOX1 (red bars) were infected with (**a**) hepatitis C virus (HCV), (**b**) severe acute respiratory syndrome-associated coronavirus 2 (SARS-CoV-2), (**c**,**d**) influenza A or B viruses (IAV, IBV), (**e**) poliovirus type 1 (PV1), (**f**) Newcastle Disease virus (NDV), (**g**) mumps virus (MuV), (**h**) Semliki virus (SeV), or (**i**) vaccinia virus (VacV). The graphs represent mean levels of infection ± S.D. (n = 3).

Naïve or PAOX1-overexpressing Huh7.5 cells were infected with vaccinia virus (VacV), severe acute respiratory syndrome-associated coronavirus 2 (SARS-CoV-2), HCV, Newcastle Disease virus (NDV), Semliki virus (SeV), mumps virus (MuV), or poliovirus type 1 (PV1). Similarly, replication of influenza A and B viruses (IAV and IBV) was studied in naïve or PAOX1-overexpressing A549 cells. In all experiments, cytopathic effects were monitored by regular observation by microscopy. In all cases, PAOX1 overexpression had no statistically significant impact on the replication of RNA and DNA viruses or their cytopathic effect (Figure 5).

### 3.7. Expression of PAOX Does Not Modulate Sensitivity of Tumor Cells to Anticancer Drugs

**Figure 6:**
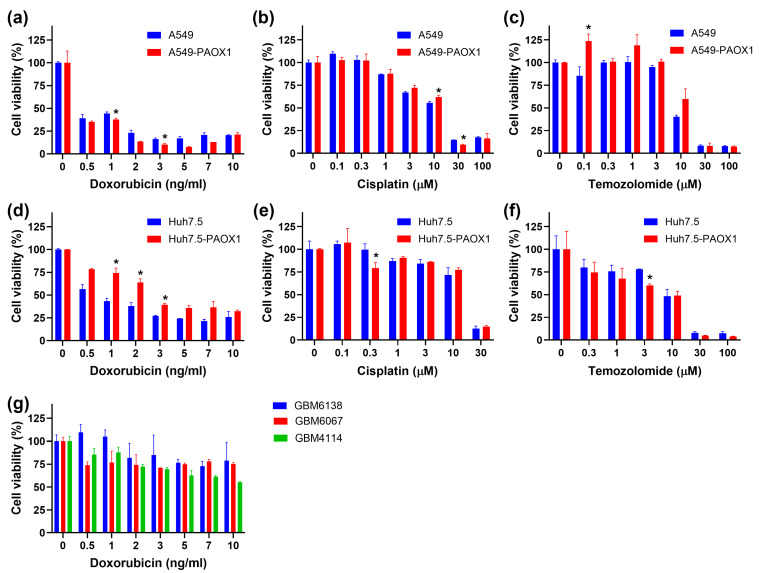
Expression of PAOX does not induce resistance of hepatocarcinoma or glioblastoma cells to genotoxic antitumor drugs. Lung carcinoma (**a**–**c**), hepatocarcinoma (**d**–**f**) or glioblastoma (**g**) cells were seeded on 96-well plates, treated with doxorubicin (**a**,**d**,**g**), cisplatin (**b**,**e**) or temozolomide (**c**,**f**) for 48 h, and viability was assessed by the MTT test. Data are shown as means ± SD. * *p* ≤ 0.05 according to a mixed ANOVA with Sidak’s post-hoc test.

As PAOX was attributed a role in conferring the resistance of cancer cells to genotoxic agents [26], we revisited this potential function of the enzyme by comparing the sensitivity of naïve and PAOX1-overexpressing A549 cells (Figure 6a–c), Huh7.5 cells (Figure 6d–f), as well as PAOX-expressing vs. non-expressing glioblastoma cells (Figure 6g) to doxorubicin, cisplatin, and temozolomide. However, no modification of the cytotoxic effects of cisplatin and temozolomide by PAOX was observed in both Huh7.5 and A549 cells (Figure 6b,c,e,f). For doxorubicin, the effect was cell-specific: PAOX1 overexpression in Huh7.5 caused a moderate reduction in sensitivity to doxorubicin (Figure 6d), whereas in lung carcinoma A549 cells, expression of PAOX1 enhanced the sensitivity of cells to doxorubicin, albeit not in a dose-dependent fashion (Figure 6a). However, statistical assessment using ANOVA supported significant differences for middle-range drug concentrations (Figure 6d). The final assessment of PAOX1’s possible role in resistance to doxorubicin was carried out in glioblastoma cells, as the GMB6138 cell line expressed high PAOX levels. Indeed, all glioblastoma cell lines displayed a high level of resistance to doxorubicin (Figure 6g), supporting the hypothesis that PAOX is a key regulator of the resistance of cells to genotoxic drugs.

## 4. Discussion

For decades, PAOX has been considered a non-rate limiting enzyme of polyamine catabolism, implying that the main pathway for degradation of spermine and spermidine involves their acetylation by SSAT, followed by rapid oxidation by PAOX, yielding spermidine and putrescine, respectively. However, in various cell lines, we were encountering very low levels of PAOX mRNA, sometimes close to the limit of detection [27,51,54]. This led us to re-evaluate the input of PAOX in polyamine catabolism by analyzing levels of *PAOX* gene transcription and quantifying enzymatic activity in cell lysates. Assessment of PAOX mRNA levels in several RNA-seq datasets obtained by our group and other laboratories revealed that this mRNA is detectible in most tumor and non-tumor cell lines, but its levels are the lowest among all polyamine-metabolizing enzymes and regulatory proteins. On average, PAOX mRNA was present at 2.1 cpm, compared to 9.4 cpm for AZIN2, 50.3 cpm for SMOX, 172 cpm for ODC, or 224 cpm for SAT1 (not mentioning 2100 cpm for GAPDH). Similar, very low levels of PAOX mRNA were shown in five human cell lines by RT-qPCR analysis. Extremely low levels of PAOX mRNA were also previously reported for clear cell renal cell carcinoma [55]. Importantly, with the exception of neuroblastoma and low-passage glioblastoma cells, we could not detect enzymatic activity, which was monitored by measuring the rate of conversion of AcSpm in cell lysates. Our data correlate with the absence of PAOX activity in colon [19] and breast [28] cancer cell lines reported by R. Casero’s group, in HEK293T cells shown by K. Porter’s group [12], as well as with very weak staining of retinal glial cells with anti-PAOX antibodies [56]. However, we should also mention that Smiraglia’s laboratory detected PAOX activity in several prostate cancer cell lines by measuring H_2_O_2_ production in cell lysates with added AcSpm using Amplex Red dye [57]. Obacan et al. also reported the detection of PAOX by western blot analysis in DU145 and LnCAP prostate cancer cells [58]. However, staining in DU145 cells was markedly less pronounced than in LnCAP cells.

We cannot exclude that PAOX activity is present in cells at levels below the limit of detection in our assay. However, the absence of detectable PAOX activity is not likely to result from technical errors, as our protocol confirmed the presence of enzymes in cells overexpressing PAOX as well as in naïve glioblastoma and neuroblastoma cell lines.

We also investigated if PAOX gene transcription is regulated during inhibition or activation of polyamine metabolism. PAOX transcription poorly responded to various stimuli, although the pattern of changes resembled patterns for two other catabolic enzymes: SAT1 and SMOX. The only exception was a marked induction in lung carcinoma A549 cells treated either with DENSpm or an exogenous spermine/spermidine mixture. We confirmed that in this case, PAOX is expressed in cells by showing that additional treatment with the PAOX/SMOX inhibitor MDL72.527 led to the accumulation of AcSpm. This suggests that under certain conditions, PAOX could be expressed and participate in polyamine catabolism, and that it is induced at the transcriptional level. However, even DENSpm failed to substantially induce PAOX in other cancer cell lines such as Huh7.5, DU145, and HeLa, implying that the effect is cell-type-specific. Indeed, Smiraglia’s team demonstrated induction of PAOX in response to DENSpm or inhibitor of the methionine salvage pathway in several prostate cancer lines (albeit) [57], while Casero’s laboratory did not find PAOX induction in the DENSpm-treated triple-negative breast cancer MDA-MB-231 cell line [28]. In hepatocyte-like HepaRG cells, DENSpm caused a decrease in PAOX mRNA, but this could be due to concomitant dedifferentiation of cells [34,54]. To date, increased expression of PAOX has been reported in response to the proinflammatory cytokine interleukin 1β (IL-1β) [59] and endoxifen, an active metabolite of estrogen receptor antagonist tamoxifen [60], while various other stimuli further decrease PAOX expression. PAOX down-regulation was previously shown during iron depletion [61], treatment with 17β-estradiol [62], or doxorubicin and concomitant genotoxic stress [26]. In the latter case, downregulation is mediated by c-Jun, JunB, and, to a lesser extent, FosB transcription factors. Hypoxia does not affect the transcription of the *PAOX* gene [56]. So, it is tempting to speculate that in the majority of cell lines and treatment conditions, the input of PAOX in polyamine catabolism is limited. According to our findings, expression of PAOX may require a substantial rise in the pool of natural (Spm and Spd) and/or synthetic (DENSpm) polyamines, although the HeLa cell line used here shows that there could be exceptions.

There is not much data on the association of PAOX with various pathologies. On the one hand, decreased levels of PAOX mRNA were reported for various tumors, including breast [25,63] and liver [64], although they did not necessarily result in changes in protein levels [64]. It contradicts our finding of increased PAOX expression in cells with high polyamine content and the widely known fact that tumors exhibit elevated pools of polyamines [1]. Upregulation of PAOX was shown in the nucleus pulposus (NP) cells during the degeneration of intravertebral disks caused by IL-1β, as well as in patients with primary myelofibrosis [65]. Notably, in the latter case, an increase in the gene copy number was also reported.

Spermine can be catabolized either by direct conversion into spermidine by SMOX or via SSAT-mediated acetylation, and the resulting AcSpm is either oxidized by PAOX or secreted from cells [16], presumably via the SLC3A2 transporter [31]. Our results from A549 cells suggest that acetylated polyamines are more readily secreted from cells than are oxidized, as SSAT and SMOX inducer DENSpm decrease the total polyamine pool by 20-fold, and polyamine oxidase inhibitor MDL72.527 only moderately prevents it (with a still five-fold reduced pool of polyamines). Moreover, the input of PAOX is lower than that of SMOX, as MDL72.527 causes more pronounced accumulation of Spm (a substrate of SMOX) than of AcSpm (a substrate of PAOX). Enhanced secretion of AcSpm during DENSpm treatment or inhibition of the methionine salvage pathway was clearly demonstrated by H. Affroni et al. of the Smiraglia laboratory [57]. So it is not surprising that acetylated polyamines can be assessed as biomarkers for pancreatic [66], hepatic [67], gastric [68], and other types of cancer. These data warrant further studies of polyamine transport and efflux, in particular.

Three sequences of protein-encoding isoforms of PAOX have been deposited in Genbank, whereas only one variant has ever been discussed in the literature. Unfortunately, the existence of alternative isoforms has never been verified in any peer-reviewed paper. Our attempts to clone these isoforms starting from mRNA yielded only the classical isoform 1, both from DU145 and A549 cells. Since the latter is already expressed at very low levels, two alternative isoforms could have very low abundancy, and here, we could not confirm them. At the same time, our study shows that even if expressed ectopically, they should not affect polyamine metabolism, as isoform 4 displays significantly diminished enzymatic activity and isoform 2 is inactive. Loss of enzymatic activity is likely to be due to the loss of amino acids that interact with the acetyl group of the substrate (such as Asn320, a homologue of Asn313 in the murine PAOX), with FAD (Lys322, a homologue of Lys315 that is involved in oxidation of its flavine group) forming pockets for substrates/cofactors or providing a negative charge required for binding/catalysis (hLeu338/mPhe331, hPhe382/mPhe375, hTyr437/mTyr430, and hSer488/mSer473) [69,70].

Previously, PAOX has been implicated in conferring resistance to genotoxic anticancer drugs such as doxorubicin [26]. The proposed mechanism of its action involved suppression of antizyme-mediated degradation of the N-terminally truncated isoform of the p73 transcription factor (ΔNp73), leading to its accumulation in cells. ΔNp73, which belongs to the p53 tumor suppressor family, lacks a transactivation domain and thus acts as an antiapoptotic protein [71]. In our previous study, we showed that resistance to doxorubicin also occurs during latent human cytomegalovirus infection, and it could be overcome with the inhibitor of polyamine oxidases, MDL72.527 [27]. Here, we revealed a correlation between PAOX activity and resistance to doxorubicin. Here, we show that PAOX could be a factor in resistance to genotoxic drugs, at least in most types of cells. However, as the effect was cell-type-specific, we cannot exclude other factors that underlie the sensitivity of tumor cells to this antitumor drug.

Biogenic polyamines regulate the replication of various DNA and RNA viruses, as reviewed in [2,46]. The abovementioned inhibitors of SMOX and PAOX (MDL72.527) have been shown to suppress replication of the Ebola virus [49], HCV [51], and SARS-CoV-2 [50]. This means that either spermine oxidase or/and acetylpolyamine oxidase is critical for virus replication. This study suggests that PAOX is dispensable for viruses, as its overexpression did not affect the replication of different RNA viruses as well as the Vaccinia virus, which belongs to a group of DNA viruses.

## 5. Conclusions

Our study suggests that PAOX may not play a significant role in polyamine catabolism in many human cell lines due to its low expression. Therefore, the flux of acetylated polyamines should be carefully re-analyzed to understand if these compounds are more likely to be exported from cells than oxidized. However, we cannot exclude the existence of other, yet unidentified, enzyme(s) responsible for the catabolism of acetylated polyamines. We also cannot exclude a more pronounced expression of PAOX in tissues compared to cell lines that would increase its input into polyamine catabolism.

## Figures and Tables

**Table 1:** Oligonucleotides used in real-time PCR.

Gene ID	Orientation	Sequence	Product Length (bp)
ODC	Forward Reverse	5′-TTGCGGATTGCCACTGATGATTCC-3′ 5′-ATCAGAGATTGCCTGCACGAAGGT-3′	186
SRM	Forward Reverse	5′-CCCGCCGAAAGTCTCTTCAA-3′ 5′-GGAAGTTCGTGCTCGGGTTC-3′	241
SMS	Forward Reverse	5′-CACAGCACGCTCGACTTCA-3′ 5′-TGCCGTTCTTGTTTGTGTAGGTT-3′	154
SSAT	Forward Reverse	5′-ATCTAAGCCAGGTTGCAATGA-3′ 5′-GCACTCCTCACTCCTCTGTTG-3′	189
SMOX	Forward Reverse	5′-GATCCCGGCGGACCATGTGATTGTG-3′ 5′-CCTGCATGGGCGCTGTCTTTG-3′	576
PAOX	Forward Reverse	5′-GTCACCGTGCCCTTAGGTT-3′ 5′-TCCCAAAGCCTATCTTCCTG-3′	103
HCV	Forward Reverse	5′-GTCTAGCCATGGCGTTAGTA-3′ 5′-CTCCCGGGGCACTCGCAAGC-3′	246
SARS-CoV-2	Forward Reverse	5′-CACATTGGCACCCGCAATC-3′ 5′-GAGGAACGAGAAGAGGCTTG-3′	128
GUS	Forward Reverse	5′-CGTGGTTGGAGAGCTCATTTGGAA-3′ 5′-ATTCCCCAGCACTCTCGTCGGT-3′	73

**Table 2:** Primers used for PAOX cloning.

Primer #	Sequence (5′-3′)
1	GACCTCCCGGCTACTCAGAA
2	GAGAAGGTGGCCCTGGTTAC
3	ATTTCTAGAATGGAGTCGACCGGCAGC
4	ATTGAATTCCTAGAGCCTGGGCCTGGG
5	TGTCACTCTCCGGAGCACT
6	GCTCCAGATCCTGTTTGCG
7	CCTAAGGGCACGGTGACG
8	TCTGTCCACGTTCTCTGTGG
9	ATTGAATTCGACCGGCAGCGTCGG
10	ATTCTCGAGGAGCCTGGGCCTGGGCT

**Table 3:** Levels of polyamines in cell lines.

Cell Line	Description	PAOX Activity	Spd Synthesis in the Presence of AcSpm (pmol/min*mg Protein)	Spd (pmol/mg Protein)	Spm (pmol/mg Protein)
Huh7.5	Hepatoma	No	<1	7.16 ± 1.14	1.53 ± 0.21
A549	Lung carcinoma	No	<1	8.34 ± 1.3	4.96 ± 0.79
DU145	Prostate carcinoma	No	<1	3.75 ± 0.52	3.51 ± 0.56
HeLa	Cervix carcinoma	No	<1	11.4 ± 1.6	24.2 ± 3.4
GBM4114	Glioblastoma	Yes	0.4 ± 0.1	7.1 ± 1.1	23.7 ± 3.8
GBM6067	Glioblastoma	Yes	13.6 ± 2.2	7.1 ± 1.1	7.0 ± 1.0
GBM6138	Glioblastoma	Yes	112.4 ± 16.8	6.1 ± 0.5	45.4 ± 3.6
SH-SY5Y	Neuroblastoma	Yes	1.7 ± 0.2	21.5 ± 3.4	178.4 ± 26.7

**Table 4:** PAOX isoforms.

mRNA		Protein		
Splice Variant	ID	Isoform	Amino Acid Residues	ID
1	NM_152911	1	511	NP_690875
4	NM_207127	2	325	NP_997010
5	NM_207128	4	486	NP_997011

## Data Availability

The raw data supporting the conclusions of this article will be made available by the authors on request.

## Supplementary Materials

The following supporting information can be downloaded at: https://www.mdpi.com/article/10.3390/cells13131134/s1, Table S1: Limits of detection (LOD) and quantification (LOQ) of polyamines by HPLC analysis, and assay reproducibility by three independent researchers; Figure S1: Representative chromatograms of polyamine quantification in the lysate of DU145 cells; Figure S2: Representative chromatograms of polyamine quantification in the lysate of HeLa cells; Figure S3: Representative chromatograms of polyamine quantification in the lysate of A549 cells; Table S2: Quantification of data, presented on chromatograms in Figures S1–S3.
