# Supplementary material for: Polyamine Catabolism Revisited: Acetylpolyamine Oxidase Plays a Minor Role Due to Low Expression
Source: Cells. 2024 Jul 1;13(13):1134. doi: 10.3390/cells13131134 (PMC11240330; doi:10.3390/cells13131134)
Supplement: Supplementary file 1 [file cells-13-01134-s001.zip › cells-3068068-supplementary.pdf]

Supplementary material to

Polyamine Catabolism Revisited: Acetylpolyamine Oxidase Plays a Minor Role due to Low Expression

Olga N. Ivanova [1], Anna V. Gavlina [1], Inna L. Karpenko [1], Martin A. Zenov [1], Svetlana S. Antseva [1], Natalia F. Zakirova [1], Vladimir T. Valuev-Elliston [1], George S. Krasnov [1], Irina T. Fedyakina [2], Pavel O. Vorobyev [1], Birke Bartosch [3,4], Sergey N. Kochetkov [1], Anastasiya V. Lipatova [1], Dmitry V. Yanvarev [1,*] and Alexander V. Ivanov [1,*]

[1] Engelhardt Institute of Molecular Biology, Russian Academy of Sciences, 119991 Moscow, Russia; martin.zenov@yandex.ru (M.A.Z.); nat_zakirova@mail.ru (N.F.Z.); pavel.gealbhain@gmail.com (P.O.V.)
[2] Gamaleya National Research Centre for Epidemiology and Microbiology of the Ministry of Russia, 132098 Moscow, Russia
[3] INSERM U1052, CNRS UMR5286, Centre de Recherche en Cancérologie de Lyon, Université Claude Bernard Lyon 1, 69008 Lyon, France
[4] The Lyon Hepatology Institute EVEREST, 69003 Lyon, France
* Correspondence: yanvarev@eimb.ru (D.V.Y.); aivanov@yandex.ru (A.V.I.)

Table S1. Limits of detection (LOD) and quantification (LOQ) of polyamines by HPLC analysis, and assay reproducibility by three independent researchers

Polyamine	LOD (nmol)	LOQ (nmol)	Standard deviation (%)
Putrescine	0,05	0,17	7,3
Cadaverine	0,05	0,18	7,5
1,6-Diaminoxehane	0,02	0,09	11,2
1,7-Diaminoheptane	0,03	0,10	12,1
Spermidine	0,01	0,04	17,7
Spermine	0,01	0,05	23,4
N^1-Acetylspermidine	0,03	0,10	10,1
N^1-Acetylspermine	0,02	0,09	8,4

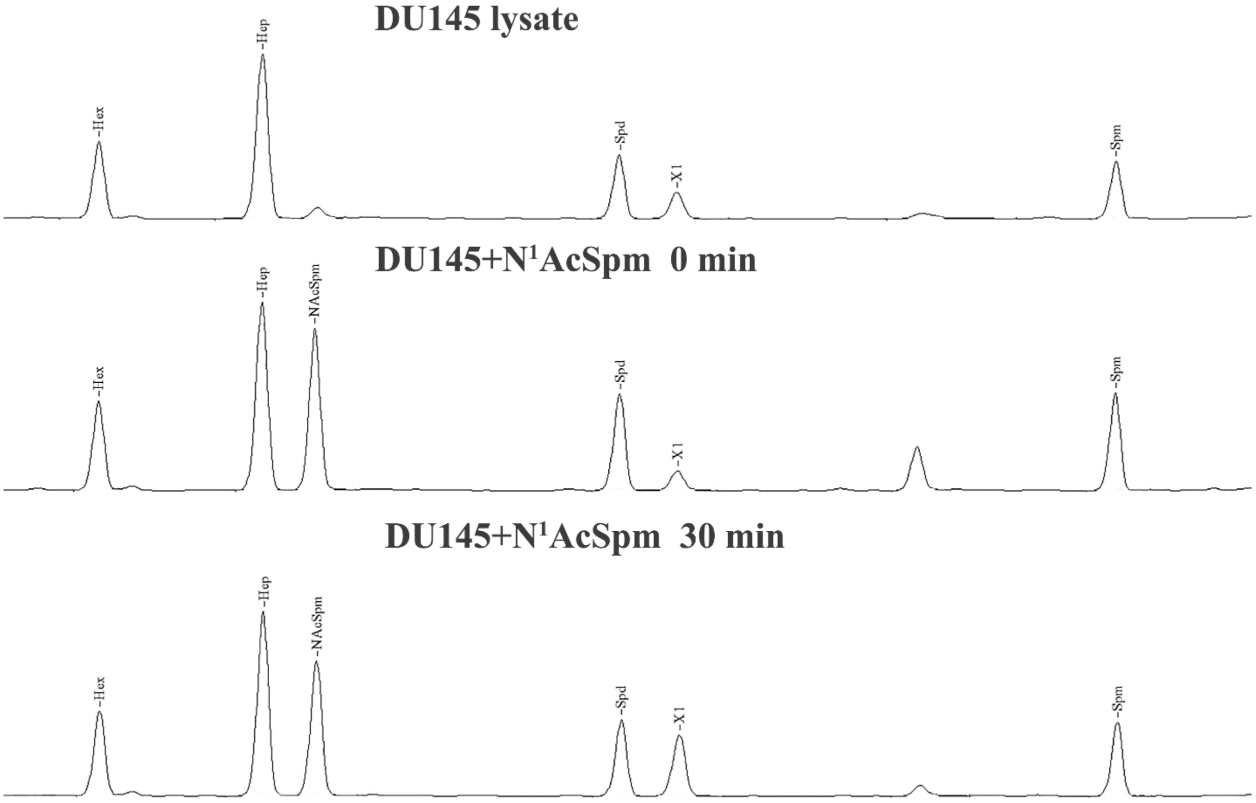

Figure S1. Representative chromatograms of polyamine quantification in the lysate of DU145 cells (upper panel) and the lysate suppelented with N^1-AcSpm prior incubation (middle panel) or after 30 min incubation (lower panel). 1,6-Diaminohexane (Hex) and 1,7-diaminoheptane (Hep) were added to reaction mixtures as internal standards. X1 denotes polyamine-nonrelated peak.

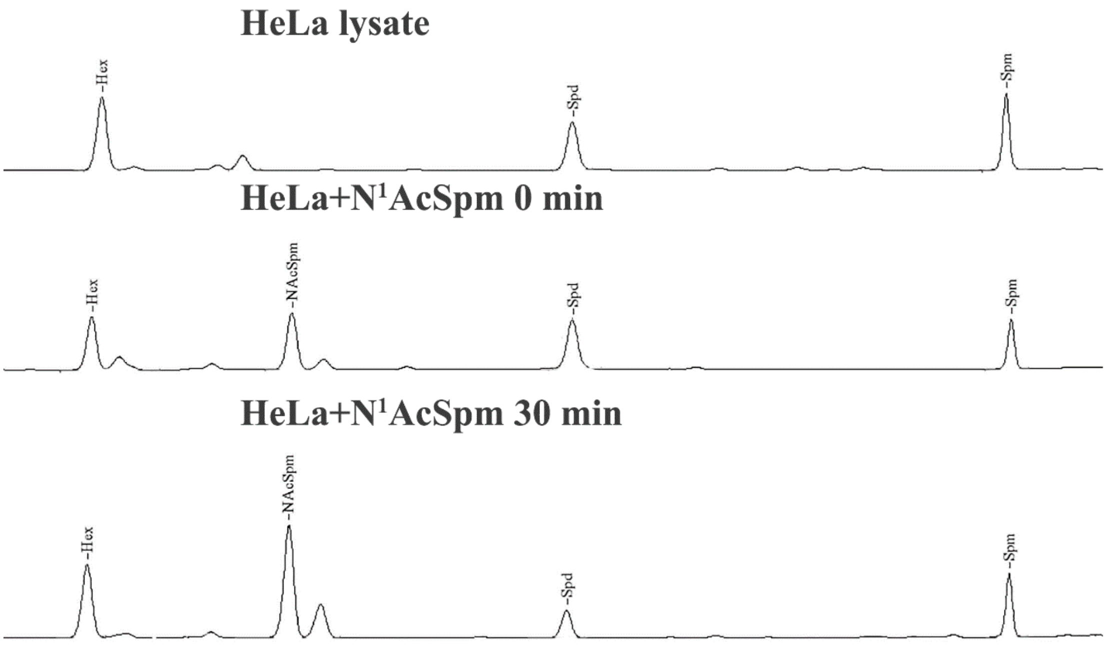

Figure S2. Representative chromatograms of polyamine quantification in the lysate of HeLa cells (upper panel) and the lysate suppelented with N^1-AcSpm prior incubation (middle panel) or after 30 min incubation (lower panel). 1,6-Diaminohexane (Hex) as added to reaction mixtures as internal standards.

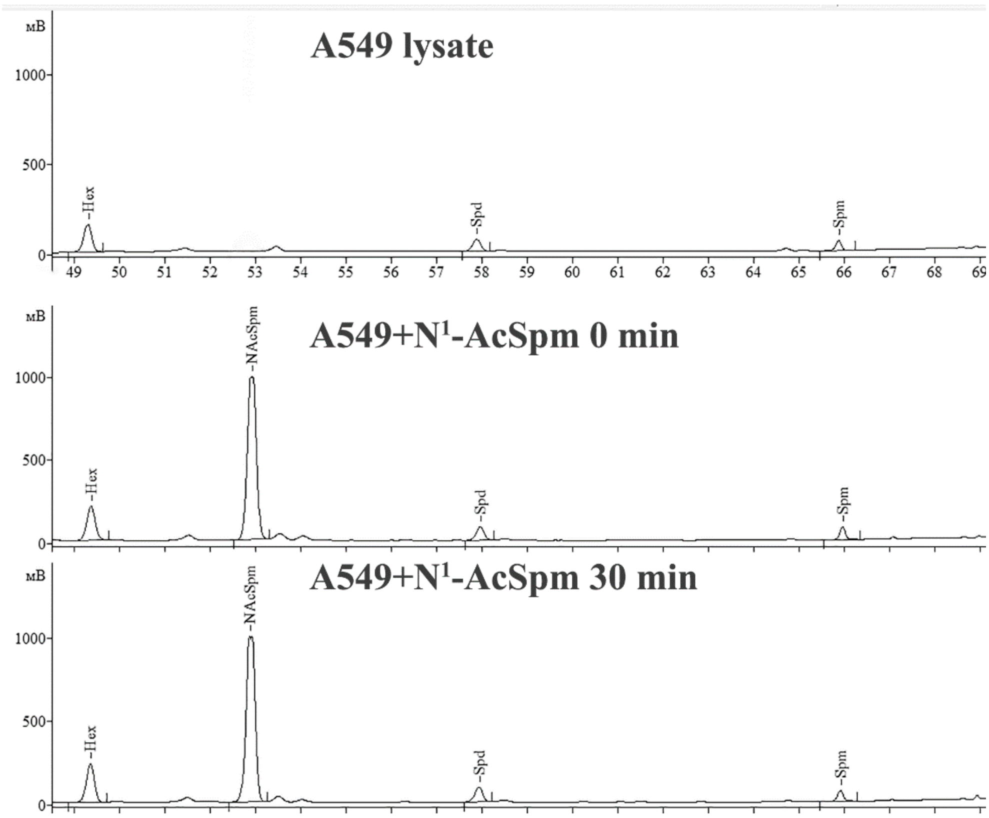

Figure S3. Representative chromatograms of polyamine quantification in the lysate of A549 cells (upper panel) and the lysate suppelented with N[1]-AcSpm prior incubation (middle panel) or after 30 min incubation (lower panel). 1,6-Diaminohexane (Hex) as added to reaction mixtures as internal standards.

Table S2. Quantification of data, presented on chromatograms S1-S3

Polyamine	N[1]-AcSpm (pmol/mg protein)	Spd (pmol/mg protein)	Spm (pmol/mg protein)
A549 lysate	<1	8,3±1,4	5,1±0,8
A549 + N[1]-AcSmp, 0 min	43,4±2,9	8,2±1,3	4,9±0,9
A549 + N[1]-AcSmp, 30 min	42,9±2,8	7,0±1,1	3,4±0,6
DU145 lysate	<1	3,8±0,5	4,0±0,6
DU145 + N[1]-AcSmp, 0 min	31,9±2,6	3,9±0,6	4,2±0,4
DU145 + N[1]-AcSmp, 30 min	28,2±2,9	3,1±0,3	3,2±0,6
HeLa lysate	<1	11,4±1,6	22,0±3,4
HeLa + N[1]-Acetylspermidine, 0 min	28,3±1,7	11,1±1,1	20,7±1,9
HeLa + N[1]-Acetylspermine, 30 min	28,2±1,3	12,1±0,9	22,9±2,4
